# Editorial: Neuro-imaging in intracerebral hemorrhage: updates and knowledge gaps

**DOI:** 10.3389/fnins.2025.1593225

**Published:** 2025-04-02

**Authors:** Marc A. Babi, Whitney Mayberry, Ahmed Koriesh, Amre Nouh

**Affiliations:** ^1^Department of Neurology, Cleveland Clinic Florida/Martin Health, Port Saint Lucie, FL, United States; ^2^Department of Neurology, Cleveland Clinic Florida, Weston, FL, United States; ^3^Cleveland Clinic Lerner College of Medicine, Case Western Reserve University, Cleveland, OH, United States; ^4^Herbert Wertheim College of Medicine, Florida International University, Miami, FL, United States

**Keywords:** intracerebral hemorrhage, neuro-imaging, computed tomography, magnetic resonance imaging, cerebral angiographies, biomarker

## Introduction

Intracerebral hemorrhage (ICH) is the most devastating subtype of stroke, estimated to account for 10%−20% of the nearly 795,000 strokes occurring annually in the United States alone (Chen et al.; Greenberg et al., 2022; Qureshi et al., 2009). The unique pathophysiology of ICH—demand rapid diagnosis and risk stratification to optimize patient outcomes given its high mortality rate of 30%−40% within the 1^st^ month (Penckofer et al.; Greenberg et al., 2022). Over the last decade, imaging has become central in the triaging, diagnosis, prognostication, acute management and prevention of ICH (Haider et al.; Penckofer et al., Qureshi et al., 2009). Computed tomography (CT) remains the first-line modality for detecting ICH owing to its widespread availability and high sensitivity (MacIntosh et al.). However, advanced CT techniques within CT—such as angiography, perfusion, and radiomics—combined with magnetic resonance imaging (MRI) sequences (e.g., susceptibility-weighted imaging and diffusion-weighted imaging) have greatly enhanced the clinicians' ability to characterize higher- risk ICH, identify patients at higher risk of clinical decline and to tailor medical and surgical interventions accordingly (Chen et al.; Hu and Sha; Zhou et al.).

This editorial summarizes recent literature findings and highlights gaps and opportunities in acute ICH imaging.

## Overview of current imaging approaches

### Non-contrast CT

NCCT remains the clinical gold standard for the initial diagnosis of acute ICH. It distinguishes between hemorrhagic and ischemic stroke with high sensitivity in the hyperacute phase. Acute intracranial hemorrhage will present as hyperdense areas on NCCT (Penckofer et al.; Qureshi et al., 2009). However, NCCT has key limitations, including lower sensitivity for subacute or chronic bleeds, and limited ability to determine underlying etiologies such as cerebral amyloid angiopathy (CAA) or vascular malformations (Greenberg et al., 2022).

### Computed tomography angiography

CTA is often performed alongside NCCT to identify underlying vascular pathologies such as aneurysms, arteriovenous malformations, or other intracranial arteriopathies (Penckofer et al.; Greenberg et al., 2022). Additionally, CTA can also detect dynamic contrast extravasation (“spot sign”)—a helpful radiographic biomarker associated with increased risk of hematoma expansion (Zhou et al.; Greenberg et al., 2022). This information can critically influence acute management, guiding intensive blood pressure reduction or consideration for additional hemostatic therapies.

### Advanced CT-based radiomics

Radiomics—an approach using advanced computational methods to extract quantitative features (shape, intensity, texture) from standard imaging—has recently emerged as a powerful tool for ICH research (Haider et al.; Zhou et al.). By analyzing subtle differences not readily apparent to the clinician, radiomics can generate predictive models for hematoma expansion and outcome. These AI and machine learning–derived features, combined with clinical markers, hold promise for tailored treatment strategies. However, a major limitation is the lack of standardization and lack of external validation.

### MRI sequences

Magnetic Resonance Imaging (MRI) offers superior tissue contrast and may detect small hemorrhages, chronic microbleeds, or underlying lesions such as cavernomas or other hemorrhagic brain lesions, more effectively than CT (Chen et al.; Zhou et al.; Penckofer et al.; Greenberg et al., 2022). In addition, several MRI-based sequences may provide additional clinical insight that guides etiology, acute and chronic management of ICH. Some of these sequences include:

1. Susceptibility-weighted imaging
  SWI is an MRI-based sequence that is particularly sensitive in identifying areas of hemosiderin or ferritin deposition. This sequence is essential in evaluating prior history of hemorrhage, such as prior microbleeds in cerebral amyloid angiopathy, subtle hemorrhagic lesions or evidence of prior silent hemorrhage.
2. Diffusion-weighted imaging
  DWI is a sequence, that although historically used mainly in acute ischemic stroke, can be an extremely helpful tool in ICH. DWI hyperintensities in acute ICH may represent hemorrhagic conversion of an ischemic infarct or ischemic changes related to peri-hemorrhagic edema. Recognizing these findings is critical to tailor appropriate diagnostic and/or therapeutic work-up in ICH accordingly.
3. Portable MRI
  Low-field portable MRI has gained increased adoption in recent years. This tool facilitates point-of-care brain imaging in critically ill patients, eliminating the risk and logistical hurdles of intrahospital transport (Shoghli et al.). Despite the current limitations of pMRI, including reduced spatial resolution and challenges in detecting smaller hemorrhages, advancements in technology or reduced costs, may soon integrate pMRI more effectively into the management of acute intracerebral hemorrhage (ICH). Several academic institutions throughout the United States have incorporated this tool in day-to-day clinical decision making.

## Summary of relevant studies

### Chronic DWI hyperintensities post-surgery

In the study by Chen et al., the investigators explored a phenomenon observed during the chronic stage of surgical management of acute ICH in which persistent diffusion-weighted imaging (DWI) hyperintensities may persist for months and occasionally mimic new pathologies such as late subacute infarcts or infections. By following patients over extended periods, the investigators concluded that these DWI signals likely represent stable imaging artifacts induced by post-surgical changes—specifically, extracellular methemoglobin “islands” that fail to be absorbed fully by the local tissue. This discovery highlights the importance of distinguishing artifactual radiographic findings vs. clinically relevant findings, and cautions clinicians to differentiate benign, stable DWI hyperintensities from progressive complications or new pathology that might otherwise warrant different interventions or additional testing.

### Automated CT segmentation

MacIntosh et al. evaluated automated segmentation of hemorrhages on non-contrast computed tomography (CT) in a large, multicenter cohort encompassing both spontaneous ICH and traumatic brain injury (TBI). The investigators deploying a deep learning algorithm trained on external datasets and validated automated volumetric estimations by matching expert manual annotations across diverse hemorrhage subtypes and hospital sites. The investigators not only highlighted the potential for standardized and rapid bleed segmentation—which is essential in both clinical triage and large-scale clinical trial recruitment—but also highlighted how automated tools might address inter-observer variability in measuring hematoma volume. This step toward more uniform quantification sets the groundwork for further AI-driven solutions in acute neuroimaging.

### Portable MRI in acute neurological conditions

In the work reported by Shoghli et al., they assessed the evolution of novel low-field (approximately 64 mT) portable MRI devices that can be used directly at a patient's bedside, thus eliminating the risks and logistical hurdles of intrahospital transport. Although resolution constraints may limit the detection of small bleeds or subtle vascular pathologies, pMRI was shown to reliably identify major acute neurological events, such as large infarcts or clinically significant intracranial hemorrhages. Importantly, the investigators highlighted the promise of these devices in resource-limited or emergent scenarios—such as rural healthcare settings. As pMRI technology evolves, quality of processed imaging improves, and become more accessible, it is likely to gain further traction in real-time assessment of neurosurgical or neurologically unstable patients.

### Radiomics and hematoma expansion

In parallel with newer technologies and innovations, two studies investigated how advanced image processing might refine risk prediction for hematoma expansion. In the study by Haider et al., the investigators compared machine learning–derived “radiomic” features of hemorrhagic lesions with traditional, visually assessed markers such as the swirl sign, black hole sign, and irregular hematoma borders. Using multicentric dataset of hypertensive ICH patients, the investigators reported that radiomic-based signatures, combined with certain clinical variables, performed significantly better at predicting hematoma expansion than either clinical variables or visual markers alone. These findings are especially relevant in the hyperacute setting of patients with acute ICH: in which early identification of patients who are at high risk of hematoma expansion, could prompt early and tailored aggressive management strategies, such as strict blood pressure control, early consideration of hemostatic therapy among other interventions. In parallel, the work by Zhou et al. focused on perihematomal edema (PHE)—a known biomarkers of secondary neuronal injury and clinical neurologic decline, and whether radiomics-based shape features of PHE itself provide additional predictive power for hematoma expansion. The investigators' results, indicated that specific shape metrics, including voxel volume and minor axis length, radscore_PHE, based, and Voxel Volume, could independently predict HE. Future studies involving longitudinal follow-up to implement and validate other imaging modalities, such as MRI, may provide valuable insights into PHE and its relationship with HE.

### Serum osmolality and outcome

Beyond radiological markers of tissue change, the work by Hu and Sha investigated the relationship between serum osmolality and in-hospital mortality among ICH patients—linking imaging findings to systemic physiological parameters. In their retrospective analysis, the investigators observed that elevated serum osmolality (≥295 mmol/L) was associated with higher in-hospital mortality. These findings, although not tied directly to imaging, highlighted how systemic states (such as intravascular volume status, renal dysfunction, dehydration, or hyperglycemia) might interplay with imaging markers of hemorrhage. The findings can further guide and screen for more prognostic markers and provide basis for disease monitoring, treatment decision-making, and prognosis improvement in ICH patients as well as guide future protocols that combine biochemical analyses with advanced imaging for further risk stratification in this patients' population.

### MRI for prognostication and future directions

In the original work by Penckofer et al., the investigators offered a comprehensive review of the expanding role of MRI in ICH. While non-contrast CT has been the gold standard and default imaging modality in the acute triage of patients with ICH, the investigators reported on the increased use of MRI sequences, particularly susceptibility-weighted imaging (SWI) to detect smaller or remote microbleeds and characterize hemorrhage age and volume with greater precision. Such refined imaging details not only help in distinguishing between primary hypertensive hemorrhages, cerebral amyloid angiopathy–related bleeds, or more distinct heterogenous etiologies, but also contribute to prognostication and preventive measures. Critically, the investigators highlight the need for future studies to assess its use in individualized clinical scenarios, such as the diagnostic value of performing serial studies and the utility of MRI in guiding specific medical and procedural interventions.

The updated American Heart Association/American Stroke Association guidelines tie together many of these emerging findings under a clinical practice umbrella. The guidelines reassert the importance of immediate neuroimaging to confirm hemorrhage, identify high risk patients based on radiographic markers and clinical signs, and delineate morphological features that predict expansion or poor outcome. Among other authors, Cordonnier et al. (2018); van Asch et al. (2010); Mayer and Rincon (2005) spotlight evolving consensus on advanced imaging—whether CTA to detect vascular abnormalities or SWI to detect microbleeds—and emphasize that these techniques must be incorporated in a structured, time-sensitive manner. By highlighting knowledge gaps—such as how frequently repeat imaging should be performed, how best to integrate imaging-derived biomarkers into treatment decisions, use of emerging AI and automated tools, and how to standardize protocols, the guidelines effectively serve as a call to action, urging clinicians and researchers to refine the integration of advanced imaging within clinical workflows.

## Conclusion

In summary, this topic highlights the remarkable strides that have been made in the neuroimaging of intracerebral hemorrhage. While non-contrast CT remains the cornerstone of immediate diagnosis, advanced platforms such as portable MRI can expand access in critical settings, and deep learning algorithms can help automate and standardize volume assessments. Radiomics has already begun to shed light on subtle morphological and textural aspects of hemorrhages and surrounding edema that can further guide clinical decisions. As the data mounts, a future direction in which ICH care is increasingly personalized, combining real-time clinical features, imaging updates and systemic biomarker monitoring, to guide precision-clinical decisions from the very early stages all the way to outpatient care. Challenge remains to validate these promising techniques in large-scale trials, ensuring cross-institutional consistency in protocols and translating this to the bedside. As the As the ICH field continues to evolve, neuroimaging innovations—ranging from advanced MRI protocols to artificial intelligence–driven data analytics—will likely form the cornerstone of the next phase of improvements in acute stroke care. By identifying patients at highest risk of expansion and guiding focused interventions, imaging stands poised to make an even larger impact in reducing the global burden of ICH.

## References

[B1] Cordonnier C. Demchuk A. Ziai W. Anderson C. S. (2018). Intracerebral haemorrhage: current approaches to acute management. Lancet 392, 1257–1268. 10.1016/S0140-6736(18)31878-6 30319113

[B2] Greenberg S. M. Ziai W. C. Cordonnier C. Dowlatshahi D. Francis B. Goldstein J. N.. (2022). 2022 guideline for the management of patients with spontaneous intracerebral hemorrhage: a guideline from the American Heart Association/American Stroke Association. Stroke 53, e282–e361. 10.1161/STR.0000000000000407 35579034

[B3] Mayer S. A. Rincon F. (2005). Treatment of intracerebral haemorrhage. Lancet Neurol. 4, 662–672. 10.1016/S1474-4422(05)70195-2 16168935

[B4] Qureshi A. I. Mendelow A. D. Hanley D. F. (2009). Intracerebral haemorrhage. Lancet 373, 1632–1644. 10.1016/S0140-6736(09)60371-8 19427958 PMC3138486

[B5] van Asch C. J. Luitse M. J. Rinkel G. J. van der Tweel I. Algra A. Klijn C. J. (2010). Incidence, case fatality, and functional outcome of intracerebral haemorrhage over time, according to age, sex, and ethnic origin: a systematic review and meta-analysis. Lancet Neurol. 9, 167–176. 10.1016/S1474-4422(09)70340-0 20056489

